# Molecular characterization of totiviruses in *Xanthophyllomyces dendrorhous*

**DOI:** 10.1186/1743-422X-9-140

**Published:** 2012-07-28

**Authors:** Marcelo Baeza, Natalia Bravo, Mario Sanhueza, Oriana Flores, Pablo Villarreal, Victor Cifuentes

**Affiliations:** 1 Departamento de Ciencias Ecológicas, Laboratorio de Genética, Facultad de Ciencias, Universidad de Chile, Las Palmeras 3425, Casilla 653, Santiago, Chile

**Keywords:** *X. dendrorhous*, dsRNA, Totivirus, Mycovirus

## Abstract

**Background:**

Occurrence of extrachromosomal dsRNA elements has been described in the red-yeast *Xanthophyllomyces dendrorhous*, with numbers and sizes that are highly variable among strains with different geographical origin. The studies concerning to the encapsidation in viral-like particles and dsRNA-curing have suggested that some dsRNAs are helper viruses, while others are satellite viruses. However, the nucleotide sequences and functions of these dsRNAs are still unknown. In this work, the nucleotide sequences of four dsRNAs of the strain UCD 67–385 of *X. dendrorhous* were determined, and their identities and genome structures are proposed. Based on this molecular data, the dsRNAs of different strains of *X. dendrorhous* were analyzed.

**Results:**

The complete sequences of L1, L2, S1 and S2 dsRNAs of *X. dendrorhous* UCD 67–385 were determined, finding two sequences for L1 dsRNA (L1A and L1B). Several ORFs were uncovered in both S1 and S2 dsRNAs, but no homologies were found for any of them when compared to the database. Instead, two ORFs were identified in each L1A, L1B and L2 dsRNAs, whose deduced amino acid sequences were homologous with a major capsid protein (5’-ORF) and a RNA-dependent RNA polymerase (3’-ORF) belonging to the *Totiviridae* family. The genome structures of these dsRNAs are characteristic of Totiviruses, with two overlapped ORFs (the 3’-ORF in the −1 frame with respect to the 5’-ORF), with a slippery site and a pseudoknot in the overlapped regions. These structures are essential for the synthesis of the viral polymerase as a fusion protein with the viral capsid protein through −1 ribosomal frameshifting. In the RNase protection analysis, all the dsRNAs in the four analyzed *X. dendrorhous* strains were protected from enzymatic digestion. The RT-PCR analysis revealed that, similar to strain UCD 67–385, the L1A and L1B dsRNAs coexist in the strains VKM Y-2059, UCD 67–202 and VKM Y-2786. Furthermore, determinations of the relative amounts of L1 dsRNAs using two-step RT-qPCR revealed a 40-fold increment of the ratio L1A/L1B in the S2 dsRNA-cured strain compared to its parental strain.

**Conclusions:**

Three totiviruses, named as XdV-L1A, XdV-L1B and XdV-L2, were identified in the strain UCD 67–385 of *X. dendrorhous*. The viruses XdV-L1A and XdV-L1B were also found in other three *X. dendrorhous* strains. Our results suggest that the smaller dsRNAs (named XdRm-S1 and XdRm-S2) of strain UCD 67–385 are satellite viruses, and particularly that XdRm-S2 is a satellite of XdV-L1A.

## Background

Mycoviruses with double-stranded RNA (dsRNA) genomes are widespread in yeasts and filamentous fungi, and unlike mammalian viruses, they do not possess an extracellular route of infection or exhibit harmful effects against their hosts [1,2]; even if some exception have been described where the mycoviruses do have a deleterious effect on their hosts [3,4]. These viruses are primarily considered as functionally cryptic genetic elements, and only in a few cases, they have been associated with a detectable phenotype in the host, such as hypovirulence or antifungal activity (killer system) [5,6]. The dsRNA viruses encoding killer systems in *Ustilago maydis* and *Saccharomyces cerevisiae* are the best characterized [7-10], and both belong to the family *Totiviridae*. This family is subdivided into the genera *TotivirusGiardiavirus* and *Leishmaniavirus,* whose members are characterized by an undivided dsRNA genome and isometric virions with no lipid or carbohydrate content, which are commonly denoted as Virus-Like Particles (VLPs) because they are not infectious [11]. The type species of the genus *Totivirus* is the *S. cerevisiae* virus L-A, found in most of the yeast strains either individually or in combination with other dsRNAs (M dsRNAs), such as occurs in the killer strains. In the 4.6-kb L-A genome, there are two overlapping ORFs: the 5’-ORF (*Gag*) encoding a major capsid protein (Gag or CP) and the 3’-ORF (*Pol*) encoding a RNA-dependent RNA polymerase (RdRp). The viral polymerase is synthesized as a fusion protein with Gag through a translational −1 frameshifting event. The genome of the M dsRNA (1.6 to 1.8 kb) only has one ORF encoding a killer toxin and self-immunity, and is called a satellite virus because it depends on the proteins encoded by L-A (helper virus) for encapsidation and replication [10].

The existence of VLPs and extrachromosomal dsRNA elements with estimated lengths of 5 (L1), 3.7 (L2), 1.4 (M), 0.9 (S1) and 0.8 (S2) kbp have been described in *Xanthophyllomyces dendrorhous* (formerly *Phaffia rhodozyma*) [12-15]. This basidiomycetous yeast has been isolated from cold climate areas around the world [16-20] and is currently one of the most promising sources of astaxanthin, a biotechnologically important carotenoid pigment [21,22]. Polymorphic dsRNA-profiles have been observed in *X. dendrorhous* in which there are strains that have zero, one, two, or four dsRNAs, and the encapsidation into VLPs have been determined for some of them [12,13,15]. In curing experiments performed with the UCD 67–385 strain, which has L1, L2, S1 and S2 dsRNAs, the concentration of L1 dsRNA is significantly increased as a consequence of the loss of S2 dsRNA (in the cured strain 385(S2)), while the amounts of L2 and S1 dsRNAs remain similar between the parental and cured strains [12,13,15]. These results suggest that the dsRNAs of *X. dendrorhous* are mycoviruses and that there exist helper/satellite systems in some strains of this yeast. However, there is no data regarding dsRNA nucleotide sequences, and the only information reported concerning relationships is derived from hybridization experiments among the dsRNAs of different *X. dendrorhous* strains, including a 5.0-kb dsRNA from *Cystofilobasidium* and the dsRNAs of strains CBS 5908 and ATCC 24203 of *X. dendrorhous*[23]. In this work, the molecular characterization of the dsRNA elements of *X. dendrorhous* was performed. The strain UCD 67–385 was selected for cloning and sequencing because it harbors almost all of the various dsRNAs reported in this yeast. Based on the molecular data obtained, the putative viral identity for dsRNAs was proposed, and the dsRNAs of different *X. dendrorhous* strains were analyzed using RT-PCR and two-step RT-qPCR methods.

## Results

### Cloning and sequencing of dsRNAs

The *X. dendrorhous* strain UCD 67–385 was selected for cloning purposes because it contains two large (L1 and L2) and two small (S1 and S2) dsRNAs, representing at least in size almost all of the various dsRNAs that have been reported in different strains of this yeast [15]. All dsRNAs used for cloning purposes and RT-PCR analyses were treated with DNase I and purified from agarose gels. The SISPA methodology [24,25] was used to clone the entire dsRNAs, but only positive results were obtained for small dsRNAs. Thus, an alternative strategy including the construction of cDNA libraries and primer walking was employed for the cloning of L1 and L2 dsRNAs, as described in the methods section. The cDNA sequences obtained from random clones were assembled, and contigs of approximately 1.6 and 1.5 kbp for L1 and L2 dsRNAs, respectively, were generated. The dsRNA-origins of the cDNAs were confirmed using RT-PCR with specific primers designed from each contig. Based on these sequences, the primer-walking experiments were performed in both directions, selecting the clones in each extension step harboring larger cDNAs by colony-PCR using vector primers. Finally, the 3’ ends of dsRNA molecules were determined using RLM-RACE as described in the methods section. This experimental strategy successfully generated the entire L2 dsRNA sequence, but some discrepant results were obtained for L1 dsRNA because several of the new sequences generated in the extension steps were not spliced at 100% with the preceding contig-assembly. These results suggested the existence of at least two molecules of L1 dsRNA with similar lengths and different sequences. This result was confirmed through comparisons with the results obtained in the sequencing of the total RNA of *X. dendrorhous* UCD 67–385 using the sequencing-by-synthesis technology (http://www.illumina.com/technology/sequencing_technology.ilmn) on the massively parallel Illumina Genome Analyzer II. Considering the sizes and nucleotide sequences obtained, five different dsRNAs were identified: L1A (4,655 bp), L1B (4,619 bp), L2 (3,962 bp), S1 (962 bp) and S2 (766 bp), which have been deposited in the GenBank database under the numbers JN997472, JN997473, JN997474, JN997475 and JN997476, respectively.

### Bioinformatic analysis and genome organization of the dsRNAs

**Figure 1:**
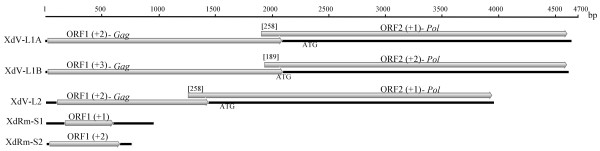
** Genome structure of dsRNAs of   *X. dendrorhous *  UCD 67–385.** The predicted ORFs and putative genes are indicated. *Gag*, major capsid protein; *Pol*, RNA dependent RNA polymerase. The ORF-frame is indicated in parenthesis. The predicted ATG-start codons for ORF 2 are indicated, and the length of the putative overlapping regions is denoted in the square brackets.

**Figure 2:**
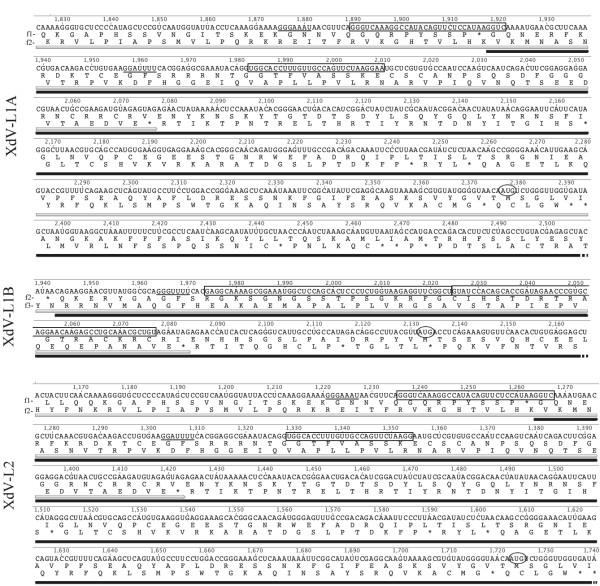
** Overlapping regions of viral dsRNAs of  *X. dendrorhous.* ** The putative slippery sites are underlined. The box indicates the predicted sequences involved in the pseudoknot structures. The ORFs for the putative CP and RdRp are indicated in gray and black, respectively.

**Figure 3:**
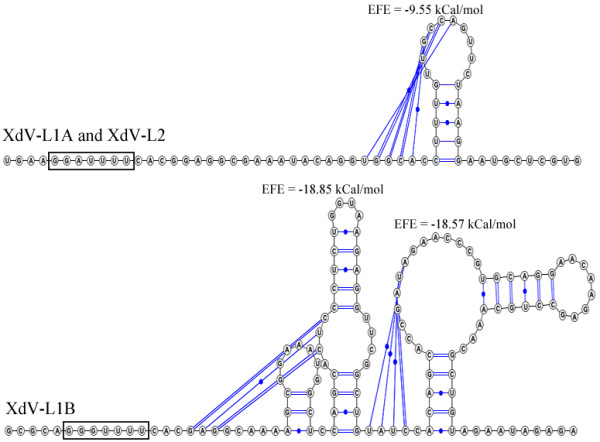
** Pseudoknot structure prediction.** The predictions were performed using a novel pseudoknot detection method called DotKnot (http://dotknot.csse.uwa.edu.au/) [56,57]. For the prediction of the estimated free energy (EFE), the method uses the pseudoknot loop entropy parameters published by Cao and Chen [58,59]. The putative slippery sites are indicated in the boxed region.

Several ORFs with lengths ranging from 108 to 600 nt were observed in the S1 and S2 dsRNAs (only the major ORFs are shown in Figure 1); a comparison against the NCBI database revealed no significant hits for the nucleotide sequences or translated ORFs. In all L1A, L1B and L2 dsRNAs, two ORFs (ORF1 and ORF2) were detected. The translated sequences of the 5’-ORF (ORF1) and the 3’-ORF (ORF2) showed homologies with the major capsid protein (CP) and an RNA-dependent RNA polymerase (RdRp), respectively, of viruses belonging to the in cursive family (Figure 1). As these results strongly suggest that the dsRNAs L1A, L1B and L2 are mycoviruses, they were designated as XdV-L1A, XdV-L1B and XdV-L2 for ** *X* ***anthophyllomyces*** *d* ***endrorhous***v**irus, whereas the small dsRNAs were designated as XdRm-S1 and XdRm-S2 for ** *X* ***anthophyllomyces*** *d* ***endrorhous* ds**R**NA **m**olecule. The nucleotide length of the predicted 5’ and 3’ untranslated regions (UTRs) of each viral dsRNA are 31 and 59 nt in XdV-L1A, 32 and 25 in XdV-L1B and 73 and 35 in XdV-L2, respectively. As shown in Figure 1, the ORFs for the putative RdRp (*Pol*) were in a −1 frame with respect to the ORFs for the putative CP (*Gag*) in the large dsRNAs. This type of genome organization is characteristic of viruses in which the *Gag* and *Pol* genes are overlapped and the RdRp is synthesized as a fusion protein with CP as consequence of a ribosomal −1 frameshifting event [26,27]. Two elements in the viral (+) RNA are necessary for the efficient induction of the frameshifting event: a) a heptanucleotide called the slippery site with the sequence X XXY YYZ (triplets indicate the pre-frameshift codons) and b) a downstream stimulatory RNA pseudoknot. If the predicted ATG-start codon for *Pol* is present as internal codon, overlapping regions of 258, 189 and 258 nt are generated in XdV-L1A, XdV-L1B and XdV-L2, respectively (Figures 1 and 2). Canonical slippery sites (X XXY YYZ) adjacent to pseudoknot structures were predicted in all three large dsRNAs (Figures 2 and 3). However, only in XdV-L1B these elements can probably induce the expression of a CP-RdRp fusion protein, as in the other two large dsRNAs both elements are located out of the overlapping region. The slippery site necessary to express the fusion proteins in XdV-LA1 and XdV-L2 can be GGAUUUU (Figures 2 and 3), a sequence slightly different from the canonical one, that can be functional as it has been demonstrated in the induction of frameshifting events in flavivirus and dianthovirus [28,29].

**Figure 4:**
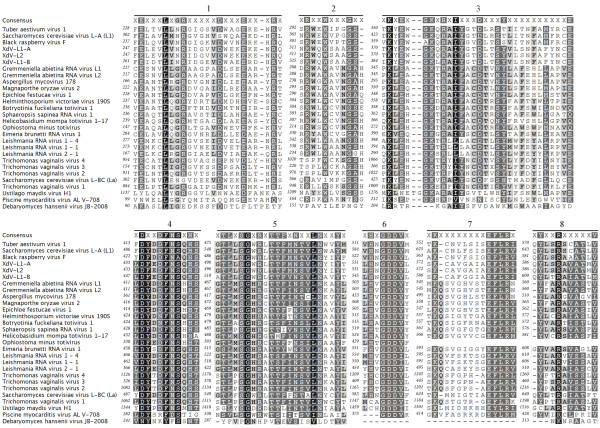
**Comparative analysis of the deduced amino acid sequences of the RdRps of *X. dendrorhous* viruses and the other 25 members of the family Totiviridae.** The consensus sequence corresponds to the residues with at least 75% conservation. The conserved domains described for the RNA polymerases are indicated with the numbers 1 to 8.

**Figure 5:**
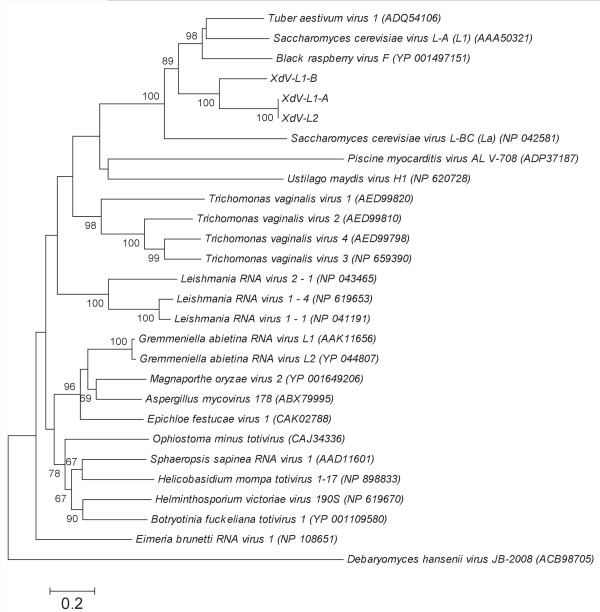
** Unrooted neighbor-joining tree based on the RdRp amino acid sequences of  *X. dendrorhous*  viruses and members of the Totiviridae family.** The optimal tree with the sum of branch lengths = 11.96693446 is shown. The bootstrap values obtained for the 500 replicates are indicated next to the branches.

The comparison of the deduced amino acid sequences of RdRps of *X. dendrorhous* viruses and those of the 25 members of the in cursive family are shown in Figure 4. All eight conserved domains, which have been previously described in viral RNA polymerases [30,31], were found in the *X. dendrorhous* viruses. These domains are almost identical among *X. dendrorhous* and *S. cerevisiae* viruses, particularly in domains 5 (SGXXXTXXXNTXXXN [X = any amino acid]) and 6 (GDD), which have been demonstrated to be essential for viral function [32,33]. In the global analysis of RdRps using the neighbor-joining method, the *X. dendrorhous* viruses were grouped together with those infecting *S. cerevisiae* and *Black raspberry* (Figure 5).

### Analysis of dsRNAs in different strains of *X. dendrorhous*

**Figure 6:**
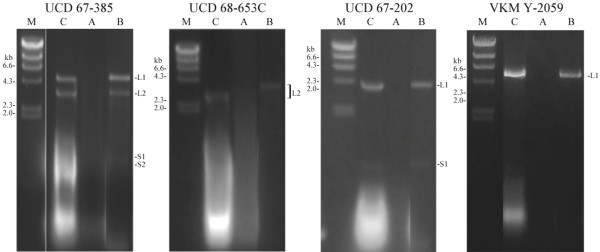
** RNase protection assays of VLPs samples extracted from different * X. dendrorhous * strains.** A: the VLPs samples digested with RNase (1 h at 37°C) after phenol–chloroform extraction; B: the VLPs samples digested with RNase (1 h at 37°C) before phenol–chloroform; C: the VLPs samples incubated for 1 h at 37°C; M, λ *Hind* III DNA marker.

To determine if the dsRNA of others strains of *X. dendrorhous* are putative mycoviruses, the encapsidation and relation to viruses of the UCD 67–385 strain were analyzed. Samples of VLPs were extracted from cultures of each strain and subjected to RNase-protection assays. As shown in Figure 6, RNase A completely degraded the dsRNAs in the VLP samples that were deproteinized by the treatment with phenol (“B” lines), in contrast to the samples not treated with phenol (“A” lines); no dsRNA bands were observed in the direct analysis of the VLP samples on agarose gels (not shown), indicating that there are no free-dsRNAs that co-purified with the VLPs. Consequently, the dsRNAs observed in the assays were protected from enzymatic digestion, implying that these dsRNAs are encapsidated. Similar to the putative viral dsRNAs, the small dsRNAs are encapsidated in strains UCD 67–385 and UCD 67–202, suggesting that the S1 and S2 dsRNAs are part of the viral system infecting these strains of *X. dendrorhous*. The L1-dsRNAs in different *X. dendrorhous* strains was examined using RT-PCR with specific primers for XdV-L1A and XdV-L1B designed in regions corresponding to the conserved domains of the RdRPs (see Table 1 and Figure 4) and the non-conserved regions of the *CP*-ORFs. The reverse-transcription reactions were performed at 50°C, and the amplicons of expected size were obtained from all L1-dsRNAs when the conserved primers were used (Table 2), but for non-conserved primers, positive results were obtained only in L1-dsRNAs of Japanese strains (UCD 67–385 and UCD 67–202), whiles no amplicon were obtained for L1-dsRNAs of strains from Russia (VKM Y-2059 and VKM Y-2786). According to these results, the L1-dsRNAs observed in the different strains of *X. dendrorhous* correspond to the two totiviruses, which are more related among strains with similar geographical origin. No amplicons were obtained from the L2 dsRNA of strain UCD 68-653C. The relative amounts of both viruses were determined using two-step RT-qPCR of the purified L1-dsRNAs with primers specific to each virus (Table 1). First, the RT reactions at 50°C were performed using the forward primers, and then the products were submitted to qPCR as described in the methods section. Several biological and technical replicates were designed to evaluate the consistency of the results. A significant amount of XdV-L1B in relation to XdV-L1A was observed in the UCD 67–385 strain, with ratio L1B/L1A of approximately 14 (Table 2). By contrast, in the cured strain 385(S2)-40, an inversion of the viral ratio was observed with a significant amount of XdV-L1A (ratio L1B/L1A = 0.4; ratio L1A/L1B = 15), suggesting an approximately 40-fold increase in relation to its parental strain UCD 67–385.

**Table 1:** Oligonucleotides used in this work

**Name**	**Position or application**	**Sequence (5’-3’)**
NBA1	Tailing of dsRNAs	(PO4)-GCAATTGTACGCCTGGAGCGC-(NH2)
NBA-1	Complementary to NBA1	GCGCTCCAGGCGTACAATTGCG
Mot4F	Conserved motif 4 of RdRp of XdV-L1A	GAGGACTTCAATAGTCAACA
Mot5R	Conserved motif 5 of RdRp of XdV-L1A	AAGTCGTCAGCCTCCACCCC
Mot6R	Conserved motif 6 of RdRp of XdV-L1A	AGACATCGTCTCCGTTGTGC
L8.2R	CP ORF of XdV-L1A	CCGTGTAGAGCTAAAATACC
SR4	CP ORF of XdV-L1A	AAGCGCATCTTCTGGGCTCA
LIVR3	CP ORF of XdV-L1B	CCTCTGCTGATCTGAAATGTT
Cap288R	CP ORF of XdV-L1B	GAAGTTTTCGCCCACAAGAG
Mot4FB	Conserved motif 4 of RdRp of XdV-L1B	GAAGATTTCAACAGTCAACATAG
Mot5RB	Conserved motif 5 of RdRp of XdV-L1B	ATGTCGTTAACCTCCAGCCC
Mot6RB	Conserved motif 6 of RdRp of XdV-L1B	GCACGTCGTCGCCGTTATG
L1AF	RT-qPCR, specific for XdV-L1A	GCGAAAATGAAGCGATGACA
L1AR	RT-qPCR, specific for XdV-L1A	TTAGTCTCCGCGCCCTTCTA
L1BF	RT-qPCR, specific for XdV-L1B	GACGAACTGATGCCCAAACA
L1BR	RT-qPCR, specific for XdV-L1B	CCGGAGACAGCUCAUUGUA
Random hexamer	Random cloning	NNNNNN
M13F	Vector	GTAAAACGACGGCCAGT
M13R	Vector	AACAGCTATGACCATG

F, forward; R, reverse.

**Table 2:** **RT-PCR and RT-qPCR from L1-dsRNAs isolated from different *X. dendrorhous* strains**

**Strain**	**Primers pairs**								**Ratio**^**a**^** L1B/L1A**
	**Mot4F Mot5R**	**Mot4F Mot6R**	**L8.2R SR4**	**LIVR3 Cap288Rv**	**Mot4FB Mot5RB**	**Mot4FB Mot6RB**	**L1BF L1BR**	**L1AF L1AR**	
VKM Y-2059	+	+	-	-	+	+	-	-	nd
UCD 67–202	+	+	+	+	+	+	-	-	nd
VKM Y-2786	+	+	-	-	+	+	-	-	nd
UCD 68-653 C*	-	-	-	-	-	-	nd	nd	nd
UCD 67–385	+	+	+	+	+	+	+	+	14 (6)
385(S2)-40	+	+	+	+	+	+	+	+	0.4 (0.2)

RT-PCR determinations were made at least 3 times for each dsRNA. *, L2 dsRNA was analyzed. +: an amplicon of the expected size; -: no amplicon; nd: not determined. ^a^, ratios were calculated from ct values obtained through RT-qPCR, the average of three biological replicates with three technical replicates are given; the standard deviations are shown in parenthesis.

## Discussion and conclusion

Previously, it has been reported that the yeast *X. dendrorhous* strain UCD 67–385 has four extrachromosomal dsRNA elements of different lengths, called L1, L2, S1 and S2. Considering the nucleotide sequences obtained in this work, there are five different dsRNAs because the L1 dsRNA corresponds to two molecules of similar lengths (not differentiable on agarose gels) and with different sequence. According to the bioinformatic analyses and comparisons against the database, the three larger dsRNAs are putative mycoviruses belonging to the family in cursive, genus in cursive, and were named XdV-L1A, XdV-L1B and XdV-L2. It was observed that all of the dsRNA-harboring strains of *X. dendrorhous* are coinfected with two or three totiviruses, a phenomenon that is not uncommon in fungi; some examples of mycovirus co-infections include the potexvirus-like M-dsRNA and SsRV-L in *Sclerotinia sclerotiorum* strain Ep-1PN [34,35]; the totivirus Hv190SV and the chrysovirus Hv145SV in *Cochliobolus victoria*[36,37]; the totiviruses SsRV-1 and SsRV-2 in *Sphaeropsis sapinea*[38]; the totiviruses ScV-L-A and ScV-L-BC in *S. cerevisiae*[39]; the partitiviruses S and F in *Penicillium stoloniferum*[40,41]; the dsRNA1-related *Fusarium poae* virus 1, and the dsRNA3-related *Helicobasidium mompa* 70 virus in the isolate V1 of the violet root rot fungus *H. mompa*[42]; the totivirus-like dsRNAs CeRV1 and CeRV2 in *Chalara elegans* (*Thielaviopsis basicola*) [43]; a mixed chrysovirus infection in a strain of the endophytic and entomopathogenic fungus *Tolypocladium cylindrosporum*[44]; and mycoviruses that contain 2–4 different segments of dsRNAs in *Fusarium graminearum*[45]. The RT-PCR analysis revealed that all L1-dsRNAs share regions encoding conserved domains of the viral polymerase, but non-conserved regions are only shared by *X. dendrorhous* strains with similar geographical origin.

The three viral dsRNAs of *X. dendrorhous* have a genetic structure that is characteristic of the totiviruses, with a putative *Pol* gene in the −1 frame with respect to the putative *Gag* gene. Thus, the viral polymerase is synthesized as a fusion protein with the capsid protein through a ribosomal frame-shifting event. The two RNA elements that are necessary for the induction of this event, a slippery site and pseudoknot structure [26,27], were revealed in the overlapping regions of each larger dsRNA. This result suggests that the larger dsRNAs are functionally independent viruses in *X. dendrorhous*. The sequence of XdV-L2 is almost identical to XdV-L1A and lacks 655 bp from the 5’-end, suggesting the possibility that XdV-L2 is a replicative intermediate of XdV-L1A. However, we believe that XdV-L2 is an independent virus that might have derived from XdV-L1A because the differences at the ends of the genomes, it is always present independent of the growth phase of the yeast [15], it is encapsidated into VLPs (according to RNase protection assays) and it is the only stable dsRNA maintained in the *X. dendrorhous* strain UCD 68-6537C. Nevertheless, a more in-depth characterization of the VLPs in relation to the existence of different types, their genomic contents and the identification of structural proteins is necessary for a better understanding of the viral system of this yeast. Although no homologies were found for the smaller dsRNAs, these dsRNAs would be part of the viral system as satellite dsRNAs because although they do not encode viral proteins, they are encapsidated and stably maintained. This proposition is supported for XdRm-S2 because, for the strain UCD 67–385, an increase of the amount of XdV-L1 dsRNAs, but not of XdV-L2, was observed in a strain cured of XdRm-S2 [15]; therefore, XdRm-S2 would be a satellite of XdV-L1A and/or XdV-L1B. Moreover, in the present work, we determined a specific increment of the XdV-L1A in the cured strain, strongly supporting that XdRm-S2 is a satellite of XdV-L1A. Contrary to the viruses of *S. cerevisiae,* the viruses of *X. dendrorhous* are extremely resistant to curing treatments. Thus, we have applied curing methodologies using different chemical agents and temperature stresses, but only the strains cured of XdRm-S2 were obtained. Finally, there were marked differences in viral content of the strains of *X. dendrorhous*, especially from different geographical origins, with strains having no viruses and strains having multiple viruses, including complex helper/viral systems. Because dsRNA mycoviruses have no extracellular route of infection, they have evolved together with their hosts; in this way, *X. dendrorhous* is a good model for virus-host coevolution studies.

## Methods

### Strains and culture conditions

The strains of *X. dendrorhous* are listed in Table 3. The yeast strains were cultured at 22°C in YM medium (0.3% yeast extract, 0.3% malt extract, 0.5% peptone) supplemented with 2% glucose. The cellular pellets for the extraction of dsRNA or VLPs were obtained after the centrifugation at 7,000 xg for 10 min of cultures in the late exponential phase of growth. *E. coli* was grown at 37°C in LB medium (1% tryptone, 0.5% yeast extract, 0.5% NaCl) supplemented with 0.1% glucose. For semisolid media, 1.5% agar (Oxoid) was used. When required, ampicillin and X-Gal were added to the media at 100 and 75 μg/ml, respectively.

**Table 3:** ** *X. dendrorhous* strains used in this work**

**Strains**	**Source**	**dsRNAs [**[15]**]**
VKM Y-2059	Flux of *Betula verrucosa*, Moscow Region, Russia.	L1
UCD 68-653C	Exudate of *Betula papyrifera*, Rainbow Lake, Kenai Peninsula, AK.	L2
UCD 67-385	Exudate of *Betula tauschii*, Shinkai, Kiso, Japan.	L1, L2, S1 and S2
UCD 67-202	*Cornus brachypoda*, Hiroshima, Japan.	L1 and S1
VKM Y-2786	Exudate of *Betula verrucosa,* Moscow Region, Russia*.*	L1
385(S2)-40	S2 dsRNA-cured strain from UCD 67–385.	L1, L2 and S1

UCD, Phaff Yeast Culture Collection, Department of Food Science and Technology, University of California Davis, Davis, USA; VKM, The All-Russian Collection of Microorganism, Moscow, Russia.

### The extraction of VLPs and the RNase protection assay

The yeast pellet (4–6 g) was washed once with distilled water and suspended in 20 ml of TBS buffer (10 mM Tris, 100 mM NaCl, 1 mM MgCl_2_, 0.1 mM EDTA, pH 7.4) supplemented with “CompleteTM Protease Inhibitor Cocktail Tablets” (Roche) according to the manufacturer’s instructions. Three grams of glass beads (0.5 mm in diameter) were added, and the samples were shaken for 30 s in a Mini beadbeater-16 homogenizer (Bio Spec, Bartlesville, USA), and subsequently cooled on ice for 1 min; the shaking and cooling steps were repeated four times. The supernatant was harvested after centrifugation at 12,000 xg for 20 min and subjected to an additional centrifugation step at 120,000 xg for 90 min (both centrifugations were performed at 4°C), and the pellet, enriched in VLPs, was suspended in 1.5 ml of TBS buffer. For the RNase protection assays, the samples were digested with 50 μg/ml RNase A for 30 min at 37°C, before and after the organic extraction with 1 volume of phenol (pH 4.0) and chloroform: isoamylic alcohol (24:1). The samples were analyzed using agarose gel electrophoresis.

### Purification of dsRNA molecules

The yeast pellet (0.1 g) was washed twice with 1 ml of TE buffer (10 mM Tris–HCl, 1 mM EDTA, pH 8.0) and suspended in 0.4 ml of the same buffer. Four hundred microliters of 0.5 mm-diameter glass beads and 400 μl of acid phenol (equilibrated with 50 mM sodium acetate, pH 4.0) were added, and the tubes were shaken for 3 min in a Mini beadbeater-16 homogenizer. The samples were centrifuged at 10,000 xg for 2 min, and the aqueous phase was extracted once with 0.4 ml of saturated acid phenol and twice with 0.4 ml of chloroform:isoamyl alcohol (24:1). The aqueous phase was transferred to an Eppendorf tube, and two volumes of isopropanol were added. The mixture was incubated at −20°C for 1 to 2 h. The RNAs were obtained after centrifugation at 14,000 xg for 20 min, dried and suspended in 20–40 μl of nuclease-free water. The samples were digested with DNase I for 1 h at 37°C and analyzed using 1% agarose gel electrophoresis in TAE buffer (2 M Tris base, 1.6 M glacial acetic acid, and 0.05 M EDTA, pH 8.0). The gels were stained with ethidium bromide (0.5 μg/ml), and each band corresponding to a dsRNA molecule was purified from the gels as previously described [46].

### Cloning of dsRNAs using the Sequence-Independent Single-Primer Amplification (SISPA) methodology

Gel-purified dsRNAs (300 ng) were ligated to NBA1 primer (250 ng) using 2 U of T4 DNA ligase and 10 U of T4 RNA ligase 2 at 37°C for 16 h. The primer-tailed dsRNAs were purified using the UltraClean^™^ 15 DNA purification kit (MO BIO Laboratories), denatured at 95°C for 10 min in the presence of 20% DMSO and 5 ng of the NBA-1 primer, and subsequently chilled in an ice water bath. The cDNAs were synthesized with 200 U of Maxima*® Reverse* Transcriptase (Fermentas International, Inc.) at 55°C for 90 min. After digestion with RNase H at 37°C for 10 min, the partial duplexes were filled in using Taq DNA polymerase at 72°C for 5 min. The PCR reactions were performed using the NBA-1 primer and the amplicons obtained were separated and purified from gels. These products were cloned using the Zero Blunt® TOPO® PCR Cloning Kit (Invitrogen). The recombinant clones were selected using colony-PCR and the M13F and M13R primers.

### Cloning of dsRNAs through cDNA library construction and primer walking

A mixture of gel-purified dsRNAs (200–300 ng) and random hexanucleotide primers (200 ng) was incubated at 95°C for 10 min in the presence of 20% DMSO, chilled in an ice water bath and incubated at 25°C for 10 min. The denatured dsRNAs were reverse transcribed with 200 U of M-MulV Reverse Transcriptase (New England Biolabs) at 42°C for 90 min, followed by digestion with 4 U of RNase H at 37°C for 90 min. The second cDNA strand was synthesized using 50 U of DNApol I (New England Biolabs) at 37°C for 120 min, followed by treatments with 2 U of T4-DNA ligase at 22°C for 30 min and 5 U of Klenow fragment at 37°C for 60 min. The cDNAs were purified on Sephadex G-50 columns (GE Healthcare), mixed with *EcoR*V-digested pBluescript SK plasmid and 2 U of T4-DNA ligase, incubated at 15°C for 16 h, and dialyzed through a 0.025-μm pore nitrocellulose disk (Millipore). The ligation mixture was used to transform *E. coli* DH5α cells using electroporation, and the white colonies that developed on the LB-amp-XGal plates were analyzed by colony-PCR with the vector primers M13F and M13R. The clones showing an amplicon of at least 200 bp were selected for plasmid purification and sequencing, and the cDNA sequences obtained were analyzed and assembled. To extend the contig-sequence obtained, divergent primers were designed at the ends of the assembly and used for dsRNA reverse-transcription; the cloning and selection of clones bearing a larger cDNA insert were performed as described above. The new obtained sequences were spliced with the preceding contig-assembly; this “extension step” was repeated until the entire dsRNA was nearly cloned. The 3’-ends of the dsRNAs were cloned using the RNA ligase-mediated amplification of cDNA ends (RLM-RACE) method [47,48]. Briefly, the oligonucleotide NBA1 was ligated to the dsRNA using a T4 RNA ligase, and the primer-tailed dsRNAs were purified using the UltraClean^™^ 15 DNA purification kit. The RT-PCR reactions were performed using the primer NBA-1 and specific internal primers for dsRNAs, and the amplicons were purified and sequenced.

### Two-step RT-qPCR

A mixture of 10 ng of dsRNA, 2 μl of DMSO and 1 μl of the forward primer (25 μM) was incubated at 94°C for 10 min. Subsequently, 1 μl of dNTPs (25 mM), 200 U of Maxima Reverse Transcriptase (Fermentas Life Sciences), 4 μl of 5X RT buffer and 9 μl of nuclease-free water were added. The mixture was incubated at 50°C for 90 min, followed by an additional incubation at 85°C for 5 min. The qPCR was performed in a MX3000P Real-time PCR Thermal cycler (Stratagene) using the 2X Sensimix SYBR kit (Bioline) according to the following conditions: volumes of 0.5, 1 or 3 μl of RT reaction products, 10 μl of Sensimix Kit, 1 μl primers mix (50 μM each), and nuclease-free water to a 20 μl final volume. The PCR cycling parameters were programmed as follows: incubation at 95°C for 10 min, followed by 40 cycles at 95°C for 15 s, 60°C for 15 s and 72°C for 15 s, and at 95, 25, 70 and 95°C for 10, 5, 1 and 1 s, respectively. The Mxpro-Mx300P v3.20 software (Stratagene) was used to calculate the Ct values, and relative quantifications were made using the 2^-ΔΔCT^ method [49,50].

### Automated DNA sequencing and data analysis

The nucleotide sequences were determined using the DNA Sequencing Kit Dynamic Termination Cycle (Amersham Biosciences Limited) and the Genetic analyzer 3100 Avant automatic sequencer (Applied Biosystem). The Macrogen INC. sequencing service was also used. The sequence data were analyzed using the Geneious Pro 5.4.5 software (Biomatters, Auckland, New Zealand).

### Phylogenetic analysis

The available amino acid sequences of the RdRp of totiviruses were retrieved from GeneBank and aligned together according to the deduced amino acid sequences of the RdRp of *X. dendrorhous* viruses employing Clustal W [51]. The evolutionary analyses were conducted using MEGA5 software [52] and the Neighbor-Joining method [53], and the evolutionary distances were computed using the Poisson correction method [54]. A bootstrapping of 500 replications was used to evaluate the robustness of the branching [55]. All positions containing gaps and missing data were eliminated.

## Competing interests

The authors declare that they have no competing interests.

## Authors' contributions

NB and MS, carried out the cloning and sequencing procedures and data analysis; OF, performed the RNase protection assays and RT-PCR experiments; PV, performed RT-PCR and RT-qPCR experiments. VC, participated in the study design; MB conceived the study and participated in its design and coordination; and MB and VC wrote the manuscript. All authors approved the final manuscript.
